# Relationship between Medium-Term Changes in Intraocular Lens Position and Refraction after Cataract Surgery with Two Different Models of Monofocal Lenses

**DOI:** 10.3390/jcm10173856

**Published:** 2021-08-27

**Authors:** Hideki Fukumitsu, Vicent J. Camps, Sara Miraflores, David P. Piñero

**Affiliations:** 1 Department of Ophthalmology, Marina Baixa Hospital, 03570 Alicante, Spain; hideki.fukumitsu@gmail.com; 2 Department of Ophthalmology, Vithas Medimar International Hospital, 03016 Alicante, Spain; 3 Department of Optics, Pharmacology and Anatomy, University of Alicante, 03690 Alicante, Spain; vicente.camps@ua.es; 4 Department of Ophthalmology, University Hospital of Torrevieja, 03180 Alicante, Spain; smirafloresg@gmail.com

**Keywords:** effective lens position, cataract surgery, intraocular lens, IOL power calculation, optical biometry

## Abstract

The aim of this prospective descriptive study was to characterize the variations of the clinical effective lens position (ELP) (considering paraxial optics and postoperative data) and the intraocular lens (IOL) position, using “eye” data gathered from a 6-month follow-up of patients who underwent uneventful cataract surgery. Patients were implanted with two different monofocal IOLs: AcrySof IQ SN60WF (Alcon) (Group 1, 247 eyes) and Akreos MI60L (Bausch & Lomb) (Group 2, 104 eyes). No significant differences were found between groups concerning spherical equivalent (SE), axial length, and clinical ELP changes, from 1 to 6 months after surgery (*p* ≥ 0.516). A more positive change in postoperative anterior chamber depth was found in Group 2, but the difference did not reach statistical significance (*p* = 0.065). No significant moderate to strong correlations were found between the changes in clinical ELP and preoperative data. The correlation between the changes in SE and clinical ELP over time was strong and statistically significant (groups 1 and 2: *r* = 0.957 and *r* = 0.993, *p* < 0.001). In conclusion, changes in refraction from 1 to 6 months after cataract surgery, with single-piece monofocal IOLs, are not clinically relevant, which correlates with the presence of good positional stability. These changes cannot be predicted preoperatively and considered in IOL power calculations.

## 1. Introduction

The world population is aging, and as a result, age-related cataracts have become a major cause of blindness worldwide [1]. Hence, cataract surgery has become the most practiced ophthalmological procedure in the world, with higher refractive expectations of patients and surgeons [2]. Although there have been advances in cataract surgery, only a fraction of patients achieve emmetropia (spherical equivalent between −0.50 and +0.50 D and astigmatism below 1.0 D) [3]. Patient dissatisfaction after cataract surgery, due to the presence of residual refractive error, could be attributed to specific factors, including inadequate calculation and selection of the intraocular lens (IOL) power, inaccurate estimation of the effective lens position (ELP), and anatomical changes that occur with surgery, affecting the predictability of the IOL power formulae [4].

Previous studies have demonstrated that uncomplicated cataract surgery induces significant modifications of several anatomical dimensions of the eye [4]. For example, several authors have reported a significant increase of the anterior chamber depth (ACD) after surgery, as well as widening of the iridocorneal angle [5,6,7]. These anatomical modifications could produce errors in ELP prediction, accounting for 22% to 38% of the total refractive prediction error [8]. Specifically, one “forward movement” of the IOL from the predicted position, according to ELP estimation, causes a myopic shift, whereas a backward displacement of the IOL results in residual hyperopia [9]. Several authors have investigated more accurate approaches for estimating ELP [10,11,12,13,14,15,16]. The influence of optic-haptic angulation in IOL positioning was shown to be significantly greater (with more variables) than edge design [16]. Likewise, the use of intraoperative ACD measurements for the estimation of ELP has shown promising results, better predicting the postoperative position of open loop IOLs and plate-haptic IOLs than preoperative ACD measurements [11,12,13].

Once implanted into the capsular bag, most IOLs experience linear forward movements during the first postoperative week followed by relatively stable IOL positions [14]. However, changes in IOL positions have been reported at longer terms, limiting the predictability of the refractive correction achieved [17,18,19]. Wirtitsch et al. [18] found that single-piece IOLs shifted significantly less postoperatively than multipiece IOLs, with a shift forward, particularly from 1 day to 1 month after surgery. Koeppl and colleagues [17] reported a decrease in ACD during the first postoperative week followed by a small increase in ACD during the first 6 months in eyes implanted with angulated 3-piece acrylic IOLs. Likewise, the largest change in the IOL position was shown to occur in long eyes, with the IOL moving back from the iris [17]. However, Klijn et al. [20] concluded in a prospective cohort study that long-term changes in refraction after cataract surgery resulted from natural fluctuations in corneal curvature rather than from IOL position shift.

The purpose of the current study was to characterize the variations of ELP and IOL positions measured with a biometry device, validated for this purpose [21] during a 6-month follow-up of the eyes of patients who underwent uneventful cataract surgery, and who were implanted with two different types of monofocal IOLs. We also investigated the relationship with residual refraction and if it could be accurately predicted from preoperative data.

## 2. Materials and Methods

### 2.1. Patients

This was a prospective, descriptive, single-center study compromising cataract patients who had undergone phacoemulsification with implantation of two different monofocal IOLs in a public hospital. Patients were recruited from January 2017 to March 2019 at the anterior segment consultation of Marina Baixa Hospital in Villajoyosa (Alicante, Spain). The research was conducted according to the tenets of the Declaration of Helsinki; written informed consent was obtained from all patients. The study was approved by the Ethics Committee of the University of Alicante.

Inclusion criteria were patients above 18 years of age, with visually significant cataracts, who underwent elective phacoemulsification surgery, and who completed a 6-month follow-up. Exclusion criteria were active ocular pathologies, previous corneal refractive surgery, corneal scars, and intraoperative complications that might have altered the stability of the IOL (capsule rupture, IOL implantation, other than “in-the-bag”, zonulodialysis, etc.). Only one eye per patient was included in the study to avoid a potentially significant level of bias from introducing data from the fellow eye of each subject, which is normally correlated. In cases of monocularly cataract surgery, the data of this eye was included. In cases of bilateral cataract surgery, one eye was selected randomly to be included in the study.

### 2.2. Ocular Examination and Follow-Up

A complete ocular examination was performed in each case within a period of two-months prior to cataract surgery. The following data from this baseline visit were collected: age, gender, central corneal thickness, Goldman intraocular pressure (IOP), subjective manifest refraction, ocular significant findings in the slit lamp examination and dilated fundoscopy, and optical biometric parameters using the Aladdin system (Topcon, Japan), which is an optical biometer and topography system combining low-coherence interferometry (super luminescent diode of 850 nm) and Placido disc topography (24 Placido rings, 6200 points covering up to 9.8 mm of cornea) to obtain a series of anatomical measurements, including axial length (AXL), ACD, pupillometry, white-to-white corneal diameter, lens thickness (LT), and anterior corneal curvature.

After surgery, all patients were evaluated the day after surgery and at 1 week, 1 month, and 6 months postoperatively. Visual acuity, subjective and objective refraction, anterior and posterior segment findings on slit lamp examination, and pseudophakic biometric data were obtained in all patients at 1 and 6 months after surgery. Only pseudophakic AXL and ACD measurements were collected during the follow-up considering that they have been to be consistent enough for research purposes [21]. It should be considered that an extremely poor consistency of the pseudophakic lens thickness measurements provided by the biometry device used in the current study was previously documented [21]. A single experienced examiner (HFM) performed all biometric measurements of the study during the morning period, between 8:30 a.m. and 3:00 p.m. In all cases, postoperative biometric measures were obtained using the pseudophakic (acrylic) mode of the software of the Aladdin system. Postoperative measurement of manifest refraction was performed in all cases by the same experienced examiner (HFM) in the same examination room, under illumination conditions simulating photopic vision. Uncorrected and corrected distance visual acuity was measured in all cases at 6 m. It should be noted that a manifest refraction was performed postoperatively to all patients, including those achieving uncorrected distance visual acuity of 0.00 logMAR.

### 2.3. Surgical Technique

On the day of surgery, the same mydriatic agents (cyclopentolate 1.0% and phenylephrine 10.0%) were used for pupil dilation in all cases. All patients had an antisepsis of the ocular surface before initiating the surgical procedure following the European guidelines. All surgeries were performed by the same surgeon (HFM) using topical or peribulbar anesthesia. Only one eye of each patient had surgery in each surgical session. 

The surgical procedure followed in all cases was composed of the following steps: creation of a 2.2-mm self-sealing clear corneal incision in the superior quadrant with a dual-beveled slit knife (Intrepid, Alcon Surgical, Inc., Fort Worth, TX, USA), manual completion of a centered continuous curvilinear capsulorhexis of 5 mm approximately, use of the chop technique for the removal of the nucleus using a 30-degree balanced tip with the Centurion phacoemulsification system (Alcon Surgical, Inc., Fort Worth, TX, USA), soft lens matter removal with a micro-coaxial irrigation/aspiration technique, and implantation of one of the two foldable acrylic IOLs of the study into the capsular bag.

Postoperatively, patients were given topical antibiotics for one week and topical steroids for 5 weeks.

### 2.4. Intraocular Lenses

One of the IOLs used in the current study was the MI60L Akreos MICS (Bausch & Lomb, Bridgewater, NJ, USA), which is a hydrophilic acrylic IOL (refractive index: 1.458) containing UV blocker. It has an aspheric optic and an overall diameter of 10.7 mm. An A-constant of 119.1 was defined by the manufacturer for this IOL. The other IOL used was the AcrySof SN60WF IQ (Alcon, Fort Worth, TX, USA), which is a hydrophobic IOL made of an acrylate/methacrylate copolymer (refractive index: 1.55) and contains a UV-blue light filter. It has an overall diameter of 13.0 mm and a biconvex-aspheric optic of 6.0 mm of diameter. An A-constant of 118.7 was defined by the manufacturer for this IOL.

The study was conducted in a public hospital; a random selection of the IOLs to be implanted was not possible. For this reason, selecting the IOLs (to be implanted) depended on the availability of one or another IOL design in the hospital inventory. Likewise, patients with significant levels of preoperative astigmatism were included in the current sample, in which a Toric IOL model would have been widely recommended. However, implanting Toric IOL models was not possible in the hospital due to public health restrictions.

### 2.5. Optical Calculations

IOL power calculations were performed in all cases involved in the current study with the SRK-T formula [22]:

(1) $$\begin{array}{c} IOL\;power\\ =\frac{1000n_{h}\left(n_{h}r_{c}-\left(n_{c}-1\right)AXL_{adj}-0.001SE_{target}(12\left(n_{h}r_{c}-\left(n_{c}-1\right)AXL_{adj}\right)+AXL_{adj}r_{c}\right)}{\left(AXL-ELP\right)\left(n_{h}r_{c}-\left(n_{c}-1\right)ELP-0.001SE_{target}(12\left(n_{h}r_{c}-\left(n_{c}-1\right)ELP\right)+ELPr_{c}\right)}, \end{array}$$

where *n_h_* is the refractive index of intraocular media (1.336), *r_c_* is the radius of curvature of the anterior corneal surface, *n_c_* is the refractive index of the cornea (1.333), *AXL_adj_* the adjusted axial length considering that is measured with optical biometry (*AXL* + 0.65696 − 0.02029*AXL*), *SE_target_* the spherical equivalent target, and *ELP_SRK-T_* the effective lens position.

The *ELP* was first estimated considering the equation recommended by the SRK-T formula to estimate *ELP* (ELPSRK-T) [22]:

(2) $$ELP_{SRK-T}=H+Offset,$$

where *H* is corneal height in mm and Offset is the offset for the specific IOL to be implanted. 

The real or clinical postoperative *ELP (ELP_clinical_)* was obtained by including the real postoperative spherical equivalent (*SE_post_*) and the IOL power implanted in the equation of the SRK-T formula. This value of ELP explains the real refractive error measured at 1 and 6 months after surgery, considering the IOL implanted and its optical power. The equation to obtain *ELP_clinical_* is as follows:

(3) $$\begin{array}{cc} ELP_{clinical}= & 1000(\frac{1}{2IOL_{power}\left(K+SE_{post}\right)})(n_{h}K+n_{h}IOL_{power}+0.001AXL_{adj}KIOL_{power}-n_{h}K\\ & \;+n_{h}SE_{post}+0.001AXL_{adj}IOL_{power}SE_{post}-n_{h}SE_{post}\\ & -{(-4n_{h}IOL_{power}\left(K+SE_{post}\right)\left(-n_{h}+0.001AXL_{adj}\left(K+IOL_{power}+SE_{post}\right)\right)\;\;+{\left(\left(n_{h}-0.001AXL_{adj}IOL_{power}\right)\left(K+SE_{post}\right)-n_{h}\left(K+IOL_{power}+SE_{post}\right)\right)}^{2})}^{1/2}) \end{array}$$

### 2.6. Statistical Analysis

Statistical analyses were performed with a commercially available software package (SPSS for Windows, version 20.0; IBM Corporation, Armonk, NY, USA). Normality of all data distributions was confirmed first, by means of the Kolmogorov–Smirnov test. The unpaired Student t test was used to analyze the differences between the two IOL groups in preoperative and postoperative data, whereas the paired Student t test was used to assess the significance of differences between postoperative consecutive visits within each group. The Pearson correlation coefficient was used to analyze the strength of the relationship between the change in ELPclinical from 1 to 6 months after surgery and changes in spherical equivalent or ACD within each group. 

## 3. Results

The sample comprised of 349 eyes (right/left eyes: 183/168) of 349 patients (male/female: 210/141) with a mean age of 73.4 years (standard deviation, SD: 7.0; range: 51 to 94 years). A total of 247 eyes (70.4%) were implanted with the AcrySof SN60WF IQ IOL (Group 1) whereas 104 eyes (29.6%) were implanted with the MI60L Akreos IOL (Group 2). Table 1 summarizes the main preoperative characteristics in both IOL groups. As shown, only statistically significant differences between groups were found preoperatively in ACD, with shorter values in Group 1 (*p* = 0.018). A total of 15 (15/247, 6.1%) and four eyes (4/104, 3.8%) had an AXL of more than 25 mm in Groups 1 and 2, respectively (*p* = 0.400).

### 3.1. Postoperative Differences between IOL Groups

Table 2 summarizes the postoperative outcomes in both IOL groups. As shown, significant differences between groups were detected in the manifest sphere and the spherical equivalent (*p* < 0.001) at 1 and 6 months postoperatively. Likewise, postoperative ACD was significantly lower in Group 1 during the follow-up (*p* < 0.001), whereas ELP_clinical_ was significantly larger in this group at 1 month (*p* = 0.025) and 6 months after surgery (*p* = 0.010). In terms of visual acuity, no statistically significant differences were found among groups in logMAR CDVA at the end of the follow-up (Group 1 0.05 ± 0.40 vs. Group 2 0.09 ± 0.21, *p* = 0.118).

### 3.2. Changes in ACD and ELP over Time in Each Group

Table 3 summarizes the changes in refraction and anatomical parameters from 1 month to 6 months after surgery in the two IOL groups evaluated. As shown, no significant differences were found among groups in the changes occurring in spherical equivalent, axial length, and ELP_clinical_ from 1 to 6 months after surgery (*p* ≥ 0.516). Furthermore, the change in postoperative ACD over time tended to be more positive in Group 2, although the difference did not reach statistical significance (*p* = 0.065).

In Group 1, changes in spherical equivalent (*p* = 0.005), ACD (*p* = 0.015), and ELP_clinical_ (*p* = 0.018) from 1 month to 6 months after surgery were statistically significant. In contrast, these changes were not statistically significant in Group 2 (spherical equivalent, *p* = 0.408; ACD, *p* = 0.194; ELP_clinical_, *p* = 0.378). Concerning changes in axial length during the follow-up, they did not reach statistical significance in either IOL group (Group 1, *p* = 0.257; Group 2, *p* = 0.527).

### 3.3. Correlation between Anatomical and Refractive Changes during the Follow-Up in Each Group

**Figure 1:**
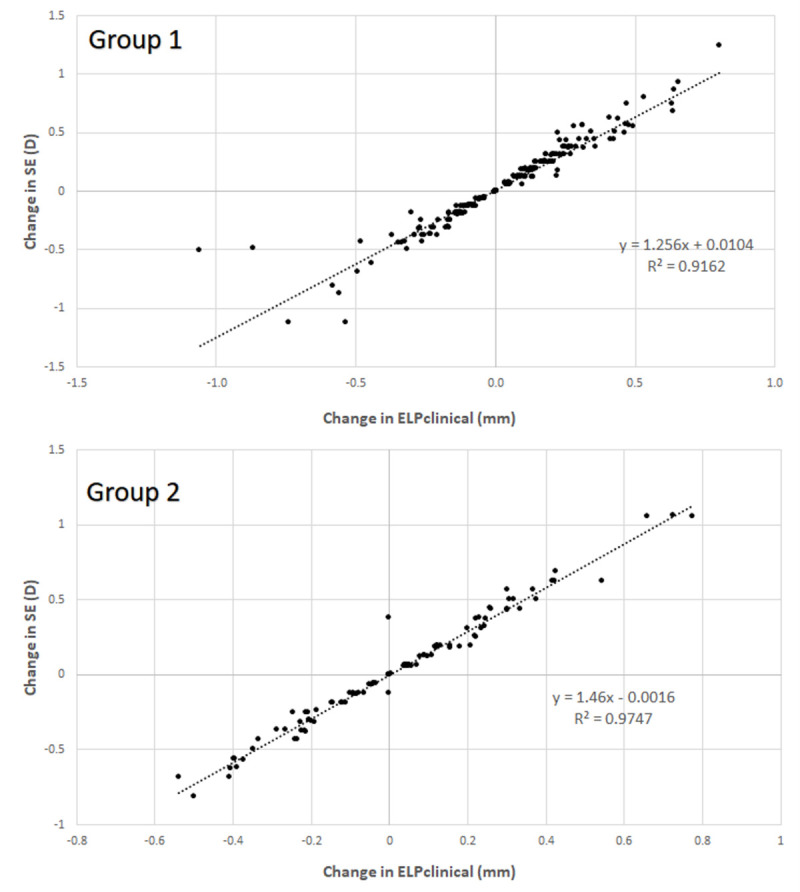
Scatter plot showing the relationship between the change in effective lens position obtained by including the real postoperative spherical equivalent and the IOL power implanted in the equation of the SRK-T formula (ELP_clinical_) and the change in spherical equivalent (SE) during the postoperative follow-up in Groups 1 and 2. The adjusting lines to the data obtained by means of the least-squares fit are shown.

In both groups, the correlation between the change in spherical equivalent and ELP_clinical_ over time was strong and statistically significant (Group 1: *r* = 0.957, *p* < 0.001; Group 2: *r* = 0.993, *p* < 0.001) (Figure 1). No significant correlation was found between the change in ELP_clinical_ during the postoperative follow-up and the change in pseudophakic ACD (Group 1, *r* = 0.098, *p* = 0.032; Group 2, *r* = −0.071, *p* = 0.575). In Group 1, no significant correlations of the change in ELP_clinical_ during the postoperative follow-up with preoperative data were found (−0.085 ≤ *r* ≤ 0.106, *p* ≥ 0.234). In Group 2, only statistically significant correlations of the change in ELP_clinical_ with preoperative IOP (*r* = 0.218, *p* = 0.029) and mean keratometry (*r* = −0.287, *p* = 0.003) were found, although they were weak.

## 4. Discussion

One of the most important issues in cataract surgery is the correct determination of the optical power of the IOL to be implanted, especially in multifocal IOLs [23]. The IOL power is determined via mathematical formulas, primarily based on paraxial optics [24,25]. In clinical practice, biometry and topography devices provide consistent and reliable measurements of anatomical measurements that are necessary to calculate the IOL power to be implanted [26,27,28]. However, the effective lens position, defined as the effective distance from the anterior surface of the cornea to the lens plane, considering a lens of infinite thinness, is still one of the main sources of error in the IOL power calculation [29]. Norrby et al. [30] found that the estimation of ELP for the calculation of the IOL power contributes 20% to 40% toward postoperative residual refractive errors. It should be noted that ELP is not the exact anatomical position of the IOL into the capsular bag, and the different IOL power formulas use different variables to estimate the ELP. Besides potential errors of prediction of ELP [4,5,6,7,8,9,10,11,12,13,14,15,16], it might experience significant changes during the follow-up once the IOL is implanted [14,17,18,19,20]. The aim of the current study was to determine the variations of IOL positions and ELP, and their relationship with residual refraction during a 6-month follow-up, in a large sample of the eyes of patients who underwent uneventful cataract surgery, with implantation of two different types of monofocal IOL.

In the current study, a low-coherence interferometry biometer was used to measure the ACD postoperatively, which has been shown to provide consistent measurements of ACD and axial length in pseudophakic eyes [21]. The ACD measurement was used to determine the IOL real position, which is not ELP, as previously mentioned. Considering the postoperative measurement of refractive error and ACD, as well as the value of the IOL power implanted, the ELP could be calculated following a procedure previously explained and described in previous studies [4]. This parameter was designated as ELP_clinical_. It was found to be significantly higher in Group 1 compared to Group 2 during the whole follow-up. This was clearly related to the significantly more anterior position of the monofocal IOL from Group 1 compared to that from Group 2, as also reported in a previous work of our research group that studied the two types of monofocal IOLs evaluated in the current series [4]. 

Concerning longitudinal changes during the follow-up, the spherical equivalent showed a small (but statistically significant) hyperopic change in Group 1, whereas no significant change in this parameter during the follow-up was observed in Group 2. This slight hyperopic change was associated with a significant reduction of postoperative ACD and an increase of ELP_clinical_. This suggests a different IOL positional behavior with the two monofocal IOLs evaluated. This may be related to differences in the IOL material and haptic design. The IOL used in Group 1 is a single-piece lens with modified L-haptics, whereas the IOL used in Group 2 is a single-piece lens with a four-point haptic design. Our results contrast with those obtained by Eom et al. [31], who compared the same two IOLs used in the current study and another three-plate IOL. These authors found that the four-plate IOL experienced significant changes in the mean ELP from 1 week to 1 month and from 3 to 6 months postoperatively, whereas changes of the other two IOLs were insignificant [31]. It should be considered that ELP was determined considering the anatomical measurements obtained from the anterior segment optical coherence tomographer in a reduced sample of eyes implanted with each IOL [31]. Wirtitsch et al. [18] found a shift forward of single-piece and multipiece IOLs, particularly from 1 day to 1 month after surgery. Koeppl and colleagues [17] reported an ACD decrease in eyes implanted with angulated three-piece acrylic IOLs during the first postoperative week, with a small ACD increase during the following 6 postoperative months. Discrepancies in the postoperative evolution of IOL position among studies seem to be related to differences in the IOL material and design, as well as in the mode of defining the position of the IOL (ELP or ACD). In our series, it can be hypothesized that the significant increase of ELP_clinical_ in Group 1 could be related to the inclusion of more “long eyes” in Group 1, with higher capsular bags and potentially higher IOL instability. However, no significant differences were found between groups in the percentage of eyes included with AXL of more than 25 mm (6.1% vs. 3.8%, *p* = 0.400), suggesting a minimal or marginal contribution of this factor to the significant change observed in ELP_clinical_ only in Group 1.

Despite the statistical significance of changes in spherical error, ACD and ELP_clinical_ in Group 1, no significant differences were found among groups when the variations were calculated and compared. Only the change in postoperative ACD over time showed a trend to be more negative in Group 2, but it did not reach statistical significance. This indicated that a slight forward movement of the IOL from Group 1 occurred, as well as a slight backward displacement of that from Group 2. These minimal anatomical changes were not significantly correlated with changes in ELP_clinical_. However, the correlation between changes in refraction and ELP_clinical_ were strong and statistically significant. This suggests that changes in cataract surgery, in the long-term, may be related to other factors, not due to positional changes of the IOL. Indeed, Klijn et al. [20] stated that long-term changes in refraction after cataract surgery are the result of fluctuations in corneal curvature rather than the consequence of an IOL position shift. In our sample, changes in spherical equivalent might be due to changes in the refraction index of the IOL or in corneal geometry [20]. Furthermore, no consistent preoperative predictive factors of this change in ELPclinical from 1 to 6 months after surgery were found, with only a very weak although statistically significant correlation of this change with preoperative IOP and mean keratometry in Group 2.

The main limitation of this study involved the limitations of the optical biometer in providing an exact measurement of the anatomical components of the eye post phacoemulsification, in which the refractive index of the intraocular media has changed. Another limitation is that it was not possible to compare different devices to confirm if the same trends were observed with other technologies. Finally, no capsular tension rings were used in the longest eyes from the current sample. It should be noted that a tendency toward higher precision in outcomes after cataract surgery, in highly myopic eyes, was reported when using capsular tension rings, although the implantation of these rings did not show a consistent effect on refractive outcomes compared with routine phacoemulsification in such eyes [32]. Despite these limitations, there are several strengths of the study, such as the large sample size and the prospective nature of the study, allowing for statistical and consistent conclusions.

## 5. Conclusions

In conclusion, changes in refraction from 1 to 6 months after cataract surgery with single-piece monofocal IOLs are not related to positional instability of the IOL, and cannot be consistently predicted in order to be considered when performing IOL power calculations. The results of this investigation could be applied to the analyzed IOLs and possibly to IOLs of the same (or similar) material or design, although more research is recommendable in the future to confirm this.

## Figures and Tables

**Table 1:** Main preoperative characteristics in the two IOL groups of the current study: Group 1 (including 247 eyes implanted with the AcrySof SN60WF IQ IOL) and Group 2 (including 104 eyes implanted with the MI60L Akreos IOL).

Mean (SD) Range	Group 1 (247 Eyes)	Group 2 (104 Eyes)	*p*-Value
Age (years)	73.5 (6.4) 51.0 to 86.0	73.1 (8.4) 59.0 to 94.0	0.691
Gender (male/female)	92/155	49/55	0.096
Corneal endothelial density (cell/mm^2^)	2302.5 (411.9) 736.0 to 3543.0	2362.9 (366.3) 1587.0 to 3461.0	0.571
Central corneal thickness (μm)	541.5 (32.3) 445.0 to 628.0	544.9 (39.4) 474.0 to 629.0	0.465
IOP (mm Hg)	16.8 (3.5) 10.0 to 29.0	16.3 (3.6) 9.0 to 25.0	0.218
Sphere (D)	0.02 (3.43) −17.50 to 6.75	0.26 (2.62) −8.25 to 4.75	0.521
Cylinder (D)	−1.44 (1.05) −8.75 to −0.25	−1.35 (1.01) −5.75 to 0.00	0.423
SE (D)	−0.70 (3.53) −17.88 to 5.88	−0.41 (2.61) −8.75 to 3.88	0.464
Axial length (mm)	23.30 (1.13) 20.59 to 27.65	23.23 (0.90) 21.40 to 25.66	0.558
ACD (mm)	3.12 (0.36) 2.24 to 3.99	3.22 (0.37) 2.09 to 3.96	0.018
Lens thickness (mm)	4.67 (0.40) 3.66 to 5.79	4.61 (0.36) 3.69 to 5.35	0.183
KM (D)	44.12 (1.57) 40.17 to 47.58	44.09 (1.30) 41.91 to 47.10	0.873
WTW (mm)	11.55 (0.36) 10.64 to 12.69	11.58 (0.39) 10.77 to 12.63	0.463
IOL power (D)	21.52 (3.13) 9.50 to 29.50	22.05 (2.35) 15.50 to 27.50	0.120
Predicted postoperative SE (D)	−0.16 (0.18) −0.82 to 0.15	−0.20 (0.16) −0.62 to 0.12	0.056

Abbreviations: SD, standard deviation; IOP, intraocular pressure; D, diopter; SE, spherical equivalent; ACD, anterior chamber depth; KM, mean keratometry; WTW, white-to-white corneal diameter; IOL, intraocular lens.

**Table 2:** Main postoperative outcomes in the two IOL groups of the current study: Group 1 (including 247 eyes implanted with the AcrySof SN60WF IQ IOL) and Group 2 (including 104 eyes implanted with the MI60L Akreos IOL).

Mean (SD) Range	1 Month Postoperative			6 Months Postoperative		
	Group 1	Group 2	*p*-Value	Group 1	Group 2	*p*-Value
Sphere (D)	0.64 (0.66) −2.00 to 2.50	0.30 (0.74) −1.75 to 2.50	<0.001	0.70 (0.67) −1.75 to 2.87	0.32 (0.73) −1.75 to 2.25	<0.001
Cylinder (D)	−1.22 (0.71) −3.50 to 0.00	−1.26 (0.84) −4.50 to 0.00	0.674	−1.23 (0.75) −3.75 to 0.00	−1.22 (0.90) −4.62 to 0.00	0.949
SE (D)	0.03 (0.57) −2.62 to 1.69	−0.33 (0.63) −2.50 to 0.88	<0.001	0.08 (0.57) −2.50 to 1.56	−0.30 (0.63) −2.56 to 1.62	<0.001
Axial length (mm)	23.22 (1.13) 20.55 to 27.52	23.14 (0.91) 21.34 to 25.48	0.560	23.22 (1.13) 20.54 to 27.50	23.13 (0.91) 21.32 to 25.48	0.422
ACD (mm)	4.61 (0.42) 3.20 to 6.23	5.36 (0.53) 3.07 to 6.33	<0.001	4.57 (0.37) 3.66 to 6.16	5.45 (0.73) 3.07 to 8.86	<0.001
ELP_SRK-T_ (mm)	Group 1 5.35 (0.48) 4.55 to 8.36			Group 2 5.30 (0.31) 4.77 to 6.41		0.268
ELP_clinical_ (mm)	5.66 (0.59) 4.23 to 9.65	5.51 (0.52) 4.12 to 6.63	0.025	5.69 (0.56) 4.46 to 8.59	5.53 (0.50) 4.21 to 6.68	0.010

Abbreviations: SD, standard deviation; D, diopter; SE, spherical equivalent; ACD, anterior chamber depth; IOL, intraocular lens; ELP_SRK-T_, effective lens position estimated considering the equation recommended by the SRK-T formula; ELP_clinical_, effective lens position obtained by including the real postoperative spherical equivalent and the IOL power implanted in the equation of the SRK-T formula.

**Table 3:** Changes in refractive and anatomical parameters during the postoperative follow-up (from 1 to 6 months after surgery) in the two IOL groups of the current study: Group 1 (including 247 eyes implanted with the AcrySof SN60WF IQ IOL) and Group 2 (including 104 eyes implanted with the MI60L Akreos IOL).

Mean (SD) Range	Group 1 (247 Eyes)	Group 2 (104 Eyes)	*p*-Value
Change axial length (mm)	−0.003 (0.040) −0.18 to 0.16	−0.007 (0.11) −0.95 to 0.21	0.611
Change ACD (mm)	−0.05 (0.30) −0.94 to 1.60	0.10 (0.63) −1.05 to 3.50	0.065
Change ELP_clinical_ (mm)	0.04 (0.24) −1.06 to 0.80	0.02 (0.26) −0.54 to 0.78	0.634
Change SE (D)	0.06 (0.31) −1.12 to 1.24	0.03 (0.38) −0.82 to 1.06	0.516

Abbreviations: SD, standard deviation; D, diopter; SE, spherical equivalent; ACD, anterior chamber depth; ELP_clinical_, effective lens position obtained by including the real postoperative spherical equivalent and the IOL power implanted in the equation of the SRK-T formula.

## Data Availability

Data available upon request due to privacy/ethical restrictions.
